# Measuring Walking Stability with a Mobile Phone in Older Adults: A Validation Study

**DOI:** 10.3390/s26072060

**Published:** 2026-03-25

**Authors:** Andisheh Bastani, Maya G. Panisset, L. Eduardo Cofré Lizama

**Affiliations:** 1 Department of Allied Health, School of Health Sciences, Swinburne University of Technology, Hawthorn, VIC 3122, Australia; eduardocofre@swin.edu.au; 2 Department of Medicine (Royal Melbourne Hospital), The University of Melbourne, Parkville, VIC 3052, Australia; maya.panisset@unimelb.edu.au

**Keywords:** balance, stability, Lyapunov, smartphone, IMU, elderly, falls

## Abstract

**Highlights:**

- Mobile phone-derived local divergence exponent (LDE) provides a valid measure of walking stability in older adults.
- In this healthy sample, walking stability did not differ between young and older adults.
- Mobile phones can be used to quantify walking stability outside the lab.
- This approach supports remote and self-assessment of walking stability in older adults.

**Abstract:**

(1) Background: The local divergence exponent (LDE) is a sensitive measure of walking stability deterioration and risk of falling in older adults. We aim to determine the validity the LDE measured using a mobile phone and to assess its ability to discriminate between healthy young and older adults; (2) Methods: 20 older adults (76.4 ± 4.6 years) and 20 young adults (29.1 ± 6.5 yrs) walked for 6 min on a 20-m walkway while wearing a research-grade inertial measurement unit (IMU) and a mobile phone placed on the sternum to record 3D acceleration data. The LDE was calculated using data from both devices for 3D, vertical (VT), mediolateral (ML), anteroposterior (AP), and norm (N) accelerations. ICC (3,1) was used to determine the validity of the mobile phone’s LDE. Mann–Whitney U tests were used to determine age-group discriminability of LDE measures; (3) Results: LDEs demonstrated excellent absolute agreement between the wearable IMU and mobile phone (ICC = 0.844). Mobile phone-derived LDEs demonstrated excellent validity relative to the wearable IMU (ICC > 0.75). No significant age-related differences in LDE were observed; wearable or mobile sensors (both *p* > 0.05); (4) Conclusions: LDEs measures obtained with a mobile phone are valid. No age group differences were identified.

## 1. Introduction

About one-third of adults over 65 years fall each year, which causes an enormous health and financial impact on the person, family, and society [1]. Falls occur due to the interaction of biological, behavioural, environmental, and socioeconomic risk factors [1]. Amongst the biological factors, assessments of physical capability deterioration (e.g., in walking and balance) are commonly used to determine falls risk and implement timely preventive interventions [2].

Measures of walking variability quantify the magnitude of step-to-step fluctuations in gait parameters and are commonly used to describe gait performance. Clinically, greater variability is often interpreted as reduced consistency of the walking pattern. However, gait variability does not directly indicate how the locomotor system responds to perturbations during walking. By contrast, gait stability reflects the capacity of the balance control system to maintain controlled locomotion in the presence of ongoing internal (e.g., neuromuscular) and external (e.g., environmental) perturbations [2]. A commonly used measure of local dynamic stability is the local divergence exponent (LDE; or maximum Lyapunov exponent) [3,4,5]. The LDE quantifies the rate at which small differences in a gait-related time series diverge over time, providing an index of sensitivity to small perturbations during walking [4,6]. Clinically, higher LDE values indicate poorer local dynamic stability and have been associated with increased falls risk, while lower LDE values following rehabilitation suggest improved walking stability [5].

To date, most studies exploring the use of the LDE as an outcome measure or as a fall predictor in the older adults have used position, velocity, or acceleration data obtained with motion capture systems (MoCap) or inertial measurement units (IMUs) [7]. Whereas MoCap systems are constrained to laboratory settings and require expertise and expensive equipment, IMUs are affordable and more accessible. The latter, however, still require a dedicated device, which presents a barrier, for example, for remote monitoring or assessment in telehealth. In this regard, the use of the mobile phone’s embedded sensors may provide an alternative to dedicated devices, allowing walking data capture using an almost ubiquitous piece of technology. Indeed, some spatiotemporal gait measures (e.g., cadence and speed) obtained with mobile phones have already been validated in the older adult population [8,9]. However, spatiotemporal measures of variability have shown poor validity [10].

Further, even accurate and reliable spatiotemporal metrics have shown limited clinical relevance in the absence of other biological measures, such as a comprehensive geriatric assessment [11]. For instance, cadence was only modestly sensitive to poor functional status [8]. In contrast, a recent study, which used a mobile phone to measure walking stability with the LDE in osteoporotic older adults, found that patients with history of falls had lower stability than those without [12]. However, to our knowledge, no study has explored the validity, reliability, and age-sensitivity of the LDE obtained with mobile phone’s sensors.

Therefore, the aims of this study were to (a) determine the concurrent validity of mobile phone-derived short-term LDE for assessing gait stability, and (b) determine the ability to discriminate between young and older healthy people (known-groups validity).

## 2. Materials and Methods

### 2.1. Participants

Contrasting age groups were used to assess known-groups validity. Participants were included in the older adult group (OG) if they (a) were older than 65 years and (b) had a Standardised Mini-Mental State Examination (SMMSE) score ≥ 25. As age-related changes in local dynamic stability have been shown to follow a nonlinear trajectory, with relatively stable values across early adulthood and a more pronounced decline emerging in midlife, rather than a continuous linear progression, we did not include a middle-age group [13]. Exclusion criteria were: (a) lacking mental capacity to consent to participate, (b) inability to speak or understand English, (c) cognitive status Modified Telephone Interview for Cognitive Status (TICS-M) < 21, and (d) having physical disabilities or uncorrected sensory impairments that prevented undertaking of tests, such as being unable to see or to walk unassisted. Participants were included in the young adult group (YG) if older than 20 and younger than 45 years, whereas exclusion criteria were the same as for the OG (except c).

### 2.2. Devices

Three-dimensional acceleration and angular acceleration data was simultaneously collected using a Movella DOT (IMU) sensor (Movella Technologies, Enschede, The Netherlands) and an iPhone SE 2020 (Apple Inc., Cupertino, CA, USA) mobile phone running on iOS 18. This device was selected due to its widespread availability and the higher completeness, correctness and consistency of sensor data acquisition within the iOS ecosystem compared to Android devices [14]. IMU wearable sensor data was recorded at 120 Hz on the sensor and extracted using the Movella DOT data exporter v.2023.6.0 (Movella Technologies, Enschede, The Netherlands), whereas mobile data was collected at 100 Hz using the Matlab Mobile app v.9.4 (MathWorks, Natick, MA, USA).

### 2.3. Procedures

**Figure 1:**
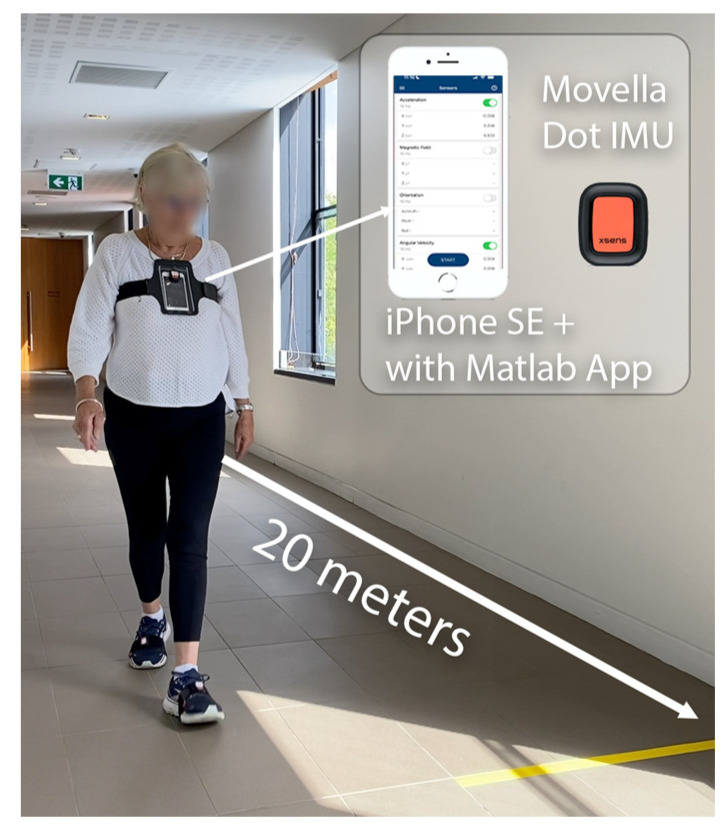
Walking space and setup for the 6MWT data collection using the mobile phone and wearable sensor (IMU).

Participants were requested to wear comfortable clothes and walking shoes for the test. The mobile phone was placed inside a pouch with a clear viewing cover, which was strapped around the chest using an elastic Velcro band, and the wearable sensor was placed over the face of the mobile phone using double-sided tape (Figure 1). Sensor location on the sternum area is based on previous research indicating that this may be a more sensitive area to detect stability deterioration in neurological populations [15]. To avoid movement artifacts, the strap was firmly tightened yet without causing discomfort when walking and breathing. Data collection with both sensors was started shortly before the commencement of a 6-min walking test (6MWT) [16] on a 20 m uncarpeted, unobstructed walkway in a physiotherapy lab or community centre. The 6MWT has been widely used to assess exercise capacity in the OG and allows capturing enough strides for an accurate estimation of gait stability using the LDE [17]. Participants were requested to walk “as far as possible” for 6 min back and forth along the walkway and stopped on the spot when the 6 min elapsed as indicated by the assessor. The total walked distance was calculated from the number of laps and remaining meters walked. Before and after completing the walking test, participants were asked to perform a brief squat to enable synchronisation of data from both sensors based on peak vertical (VT) acceleration.

### 2.4. Data Analyses

Three-dimensional acceleration data and angular acceleration from both devices, wearable sensor and mobile phone, were first cropped using VT acceleration peak at the start and end of the trial. Peak angular acceleration in the transverse plane was used to identify turns and remove 1.5 s of data before and after. For each lap, peak VT acceleration was used to identify the beginning of a gait cycle and mediolateral (ML) acceleration to determine if data corresponded with a left (positive) or right (negative) stride. Walking data, starting and finishing with a left stride for each lap was then stitched to extract the middle 150 strides [4]. For a better comparison between the short-term LDEs calculated from sensors collecting at different sample rates, wearable sensor data was down sampled to 100 Hz to compare with mobile phone data. The short-term LDEs (λ_0–0.5_ strides) were then calculated over the n strides ×100 samples normalised three- dimensional (3D), VT, ML, anteroposterior (AP) and norm (N) timeseries utilising the Rosenstein algorithm [18]. For all state-space reconstructions we used a fixed *m* = 5 (embedded dimensions) and *t* = 10 (time delay in samples), which is consistent with common practice in gait LDE studies [7,15,19]. Higher values indicate greater instability.

### 2.5. Statistics

All analyses were conducted using IBM SPSS Statistics v30.0.0.0 (IBM Corp., Armonk, NY, USA). To assess agreement between the mobile phone and wearable sensor measurements for short-term LDE outcome, we calculated Intraclass Correlation Coefficients (ICC) using a two-way mixed-effects model for absolute agreement (single measures), as recommended for device validation. ICC values were interpreted as poor (<0.50), moderate (0.50–0.75), good (0.75–0.90), and excellent (>0.90) [20]. Bland–Altman analyses were conducted to assess bias and limits of agreement (mean difference ± 1.96 SD) between devices, both overall and for each direction (VT, ML, AP, N, 3D) [21].

To evaluate age-group discriminability of short-term LDE measures, we performed Mann–Whitney U tests comparing YG and OG for each direction and sensor. Bonferroni correction was implemented to adjust for multiple comparisons. Visual differences were illustrated using clustered box plots (Direction × Group) for each sensor, annotated with significance markers based on Mann–Whitney results [22]. Mann–Whitney U tests was also performed to compare walking speed between YG and OG separately for each sensor.

Additionally, a linear mixed model was used to examine group differences across directions while adjusting for speed as a covariate, with Group, Direction, and Group × Direction as fixed effects. All analyses were repeated for both sensors (Mobile phone and wearable sensor) and for each direction separately [23].

## 3. Results

### 3.1. Participants

Twenty healthy older adults (2 Male, 18 Female) and twenty healthy young adults (8 Male, 12 Female) were recruited for this study. Demographics are presented in Table 1. All participants read and signed an informed consent prior participation. This study was approved by Swinburne University of Technology Ethics Committee (SUHREC 20237514-16788).

### 3.2. Validity

The ICC values, 95% confidence intervals, and *p*-values for short-term LDE between the two wearable and mobile phone sensors are summarised in Table 2. Short-term LDE measures obtained from the mobile phone showed excellent agreement [24] with the wearable sensor reference system (ICC > 0.75). The ICC values for each direction are also provided in Table 2.

### 3.3. Agreement

**Figure 2:**
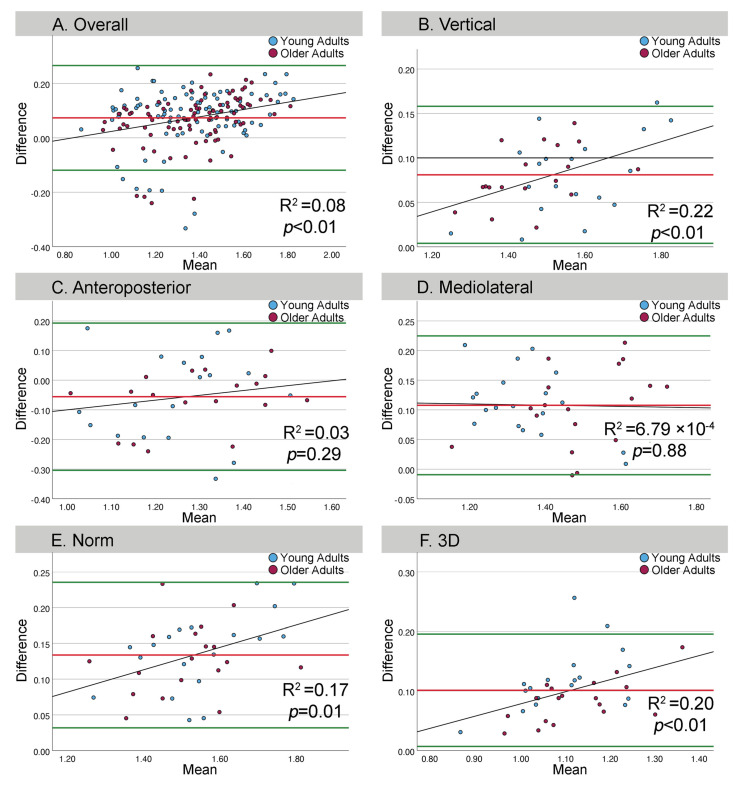
(**A**) Bland–Altman plot showing the overall agreement for all short-term LDEs between the wearable and mobile sensors. Bland–Altman plots showing agreement between short-term LDE in the vertical (**B**), anteroposterior (**C**), and mediolateral (**D**) directions as well as for the acceleration norm (**E**) and 3D (**F**). In each of the plots, horizontal green solid lines indicate 95% limits of agreement, whereas bias is indicated by solid red lines. Blue and purple circles represent data points from young and older participants, respectively. The black solid line indicates the linear regression fit using Bland–Altman regression.

Direction specific Bland-Altman plots demonstrated direction dependent agreement between the wearable sensor and the mobile phone sensors (Figure 2). In the VT direction, a positive bias of 0.081 was observed, with 95% limits of agreement (LoA) from 0.004 to 0.158, indicating good agreement and systematically higher values recorded by the wearable sensor compared with the mobile phone. In the ML direction, the bias was 0.107, with LoA ranging from −0.003 to 0.224. Although the lower LoA slightly crossed zero, the overall bias remained positive, reflecting higher wearable sensor measurements and no evident heteroscedasticity on visual inspection.

In the AP direction, a small negative bias (−0.055) was observed, with wider LoA (−0.304 to 0.192), indicating reduced agreement and a tendency for the mobile phone to yield higher values than the wearable sensor in this direction. The N direction exhibited the largest positive bias (0.133), with LoA entirely above zero (0.031 to 0.235), indicating a consistent systematic difference between sensors and relatively tight agreement. For the 3D measure, the bias was 0.101, with LoA from 0.007 to 0.195, suggesting good overall agreement, with higher values obtained from the wearable sensor on average.

Overall agreement was direction-dependent, with the tightest agreement in VT and N and the weakest in AP, which showed the widest LoA (Figure 2; Table 3). Proportional bias was assessed by regressing Bland–Altman differences (wearable − mobile) on the corresponding means ((wearable + mobile)/2), where a non-zero slope indicates proportional bias. Overall, the regression showed proportional bias (B = 0.134, 95% CI 0.068–0.201, *p* < 0.001; R^2^ = 0.081), indicating the wearable reads progressively higher than the mobile as short-term LDE increases.

By direction, proportional bias was evident in VT (B = 0.131, 95% CI 0.047–0.216, *p* = 0.003; R^2^ = 0.222; F(1,35) = 9.998), N (B = 0.156, 95% CI 0.037–0.276, *p* = 0.012; R^2^ = 0.168; F(1,35) = 7.070), and 3D (B = 0.202, 95% CI 0.060–0.344, *p* = 0.007; R^2^ = 0.197; F(1,34) = 8.354), showing an increasing positive wearable–mobile difference at higher LDE magnitudes. In contrast, no proportional bias was detected in ML (B = −0.011, 95% CI −0.149–0.128, *p* = 0.878; R^2^ = 0.001) or AP (B = 0.163, 95% CI −0.144–0.471, *p* = 0.289; R^2^ = 0.032). Collectively, proportional bias is direction-dependent (present in VT, N, and 3D, absent in ML and AP) and should be considered when interpreting device interchangeability across directions.

### 3.4. Discriminability

A Mann–Whitney U test showed that walking speed was higher in YG than in OG (U = 81.0, Z = −2.735, exact two-tailed *p* = 0.006; r = 0.45). Mean ranks were higher for YG (23.74) than OG (14.00), and median [IQR] speeds confirmed this pattern (YG: 1.49 [1.26–1.68] m/s; OG: 1.27 [1.09–1.34] m/s), indicating a clear shift toward faster walking in the YG. Given that walking speed is a major confounder for gait biomechanics, all subsequent comparisons were adjusted for speed.

Overall, no between-group differences in short-term LDE were observed for the wearable sensor (Mann–Whitney U = 4104.0, Z = −0.47, *p* = 0.639). OGs showed only slightly higher values than YGs participants (median [Q1–Q3]: 1.44 [1.23–1.58] vs. 1.42 [1.24–1.56]). Similarly, no significant between-group differences were found for short-term LDE derived from the mobile sensor (U = 3976, Z = −0.70, *p* = 0.482), with comparable values between older and younger participants (median [Q1–Q3]: 1.35 [1.22–1.48] vs. 1.32 [1.17–1.49]).

Direction-specific analyses of wearable IMU data identified significant between-group differences in short-term LDE in the VT (U = 101, Z = −2.12, *p* = 0.033) and ML (U = 67, Z = −3.16, *p* = 0.002) directions. YG participants exhibited higher VT LDE than OGs (median [Q1–Q3]: 1.60 [1.51–1.68] vs. 1.49 [1.38–1.59]), whereas OGs exhibited higher ML LDE than YGs participants (median [Q1–Q3]: 1.51 [1.47–1.69] vs. 1.37 [1.31–1.47]). No significant differences were observed in the AP, N, or 3D directions (all *p* > 0.05).

Direction-specific analyses of mobile sensor data showed the same pattern, with significant between-group differences in the VT (U = 94, Z = −2.34, *p* = 0.019) and ML (U = 72, Z = −3.00, *p* = 0.003) directions. OGs demonstrated lower VT LDE than YG participants (median [Q1–Q3]: 1.42 [1.32–1.50] vs. 1.51 [1.43–1.63]) and higher ML LDE (median [Q1–Q3]: 1.47 [1.34–1.51] vs. 1.27 [1.20–1.35]). No statistically significant between-group differences were observed in the AP, N, or 3D directions (all *p* > 0.05).

**Figure 3:**
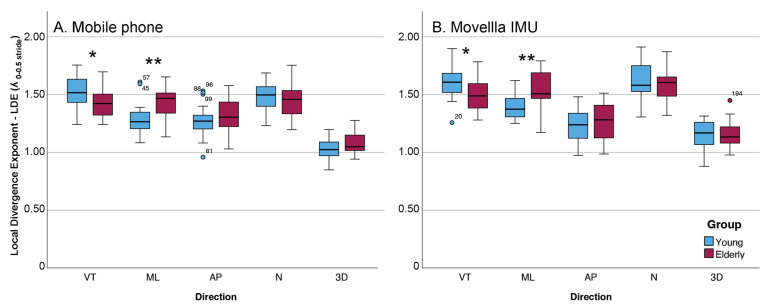
Distribution of short-term LDE across movement directions for young and older participants measured using the mobile phone (**A**) and Movella IMU wearable (**B**) sensors. Boxes represent interquartile ranges, horizontal lines indicate medians, and whiskers denote data spread. (* *p* < 0.05, ** *p* < 0.01).

Mixed-model ANCOVA adjusted for walking speed showed no main effect of Group on short-term LDE (F(11,74) = 0.279, *p* =0.598, partial η^2^ = 0.002), but a main effect of Direction (F(41,74) = 74.942, *p* < 0.001, partial η^2^ = 0.633). The Group × Direction interaction was significant (F(41,74) = 4.705, *p* = 0.001, partial η^2^ = 0.098), indicating that between-group differences depended on movement direction. In simple-effects analyses (Group within each direction; speed-adjusted), OGs showed higher short-term LDE than YGs in the ML direction (F(1,34) = 5.008, *p* = 0.032, partial η^2^ = 0.128), with no group differences in VT (F(1,34) = 1.883, *p* = 0.179), AP (F(1,34) = 0.030, *p* = 0.863), N (F(1,34) = 0.028, *p* = 0.869), or 3D (F(1,34) = 0.140, *p* = 0.711). After Bonferroni correction for five comparisons (α = 0.01), the ML simple effect did not remain statistically significant, suggesting a direction-specific trend in ML that does not withstand adjustment for multiple testing (Figure 3).

## 4. Discussion

The aims of this study were (i) to determine the validity of mobile phone–derived LDE relative to a gold-standard wearable sensor, and (ii) to evaluate the ability of mobile phone LDE to discriminate between age groups. Overall, we found high agreement between mobile-derived and research-grade wearable LDE, supporting the reliability of the mobile approach. However, unlike previous studies, we did not observe significant age-group differences in LDE between older and younger adults. This work was motivated by the need for accessible, low-cost tools to assess and monitor walking stability in older adults, who are at increased risk of falls when balance control is insufficient to maintain dynamic stability [19].

To our knowledge, this is the first study examining the concurrent validity of mobile phone–derived LDE as a measure of walking stability against research-grade wearable IMUs. In this study, mobile phone LDE (directional, N, and 3D) showed high agreement with the wearable reference and excellent reliability, supporting the feasibility of using smartphones to quantify walking stability in both older and younger adults.

This has practical relevance because smartphones offer an accessible, low-cost platform for assessing walking stability outside specialist laboratories. Importantly, smartphone use is common among older Australians: 95% of adults aged 65–74 and 84% of those aged 75+ report using a mobile phone to go online [25]. Consequently, mobile phone–derived LDE has potential to support more frequent, routine (self-)assessment of walking stability in everyday clinical and community settings. However, broader implementation will require standardised assessment protocols to minimise environmental effects on walking performance. For example, using a consistent test such as the 6MWT (as in this study), with standardised instructions and setting, may improve consistency and reproducibility—particularly if smartphone LDE is to be used to estimate falls risk or monitor responses to preventive and rehabilitation interventions. Finally, stability estimates derived from LDE are known to be influenced by methodological choices (e.g., algorithm, data length/number of strides, and parameter selection). These factors were not a focus of the present work; however, we followed prior recommendations and used commonly adopted parameter settings.

To enable a fair comparison between mobile phone and wearable IMU- derived LDE, the wearable IMU acceleration was down sampled to match the phone’s sampling rate (100 Hz), minimising potential bias due to differences in sampling frequency. Notably, prior work suggests that even larger sampling differences (e.g., 100 vs. 200 Hz) have minimal impact on LDE estimates [26]. We adjusted the discriminability analyses for walking speed because it is a major confounder when comparing gait biomechanics between groups, including LDE [27]. This is particularly relevant given that many young–old comparison and clinical studies have used treadmill walking to control speed [7]; however, treadmill walking may introduce its own confounding because overground and treadmill conditions can yield different LDE values [28]. In this study, we therefore prioritised an overground assessment aligned with established clinical protocols (e.g., the 6MWT) while statistically accounting for walking speed.

Our second aim was to evaluate whether LDE derived from both the wearable IMU and the mobile phone (directional, norm, and 3D) could discriminate between older and younger adults. Overall, we observed no significant between-group differences, which contrasts with most prior young–old comparisons [3,7]. Although not statistically significant, we noted a trend toward higher (less stable) LDE in the ML direction in older adults, consistent with previous reports [29]. In contrast, the VT direction showed an opposite trend in our sample. One possible explanation is between-directions compensation, whereby changes in one axis are offset by others; this is supported by the absence of a clear trend in the 3D and N LDE measures. Differences in walking speed may also have contributed to these findings. Although analyses were adjusted for speed, residual confounding cannot be excluded because the effect of walking speed on LDE remains uncertain. Previous studies have reported contradictory findings, including lower divergence at slower speeds, no significant effect, and method-dependent effects [19,30,31]. In addition, most of this evidence derives from treadmill walking, which may not translate directly to overground gait [19,30,31]. Therefore, the absence of overall group differences should be interpreted cautiously.

Using LDE, several studies have reported lower dynamic stability in fallers than non-fallers [32], and associations between reduced stability and prior fall history. LDE has also shown some utility for falls prediction, particularly when combined with clinical scores and other gait measures, although predictive performance is typically modest when used as a stand-alone metric [33,34,35]. However, the evidence base remains limited by the small number of prospective studies and substantial methodological heterogeneity (e.g., sensor placement, preprocessing, state-space reconstruction, and parameter selection). In this context, our findings support the feasibility of using mobile phones to quantify LDE, enabling larger-scale studies to examine stability changes in older adults and their relationship with falls.

Impaired balance control and reduced walking stability, which contribute to increased fall risk, have also been reported in other populations such as multiple sclerosis [36], Parkinson’s disease [37], and stroke [38]. By supporting the validity of mobile phone–derived LDE as an index of walking stability, this work provides a foundation for developing accessible tools for frequent (self-)assessment and remote monitoring of gait stability, and for using LDE as an outcome measure in preventive and rehabilitation programs, as well as (non-) pharmacological interventions.

*Limitations*: We placed the phone on the sternum rather than at the more common lumbar location, which is often considered a proxy for centre of mass [39], reflecting the overall biomechanics of gait. Indeed, lumbar placement has been shown to be the “minimal-optimal” placement for assessing global parameters of treadmill running in healthy adults using spatiotemporal measures [40]. Non-central sensors (bilateral ankles) were required, however to detect gait asymmetry [40]. However, we previously found, using nonlinear measures, that sternum placement was more sensitive to deficits in dynamic stability in non-disabled people with multiple sclerosis [15]. We suggest that may be more practical for clinical populations and older adults, because it allows easier positioning, checking, and secure adjustment for testing. In addition, sternum placement provides a more consistent sensor orientation across participants and may reduce uncontrolled motion artefact compared with, e.g., pocket carriage. We acknowledge that chest placement is less representative of typical everyday phone use and may not be conducive to passive monitoring applications. However, we believe that a dedicated pouch or holder should be considered to facilitate rapid daily-life assessments, ensure signal stability and, ultimately, provide a more accurate and clinically meaningful estimation of walking stability.

We also used fixed LDE parameters. Although some group-comparison studies select parameters based on sample median values, fixed settings are unlikely to affect the present validity/reliability analyses because they were applied consistently across repeated measures; moreover, fixed-parameter approaches have also detected significant between-group differences in prior work (*m* = 5 for embedded dimensions and *t* = 10 for time delay in samples) [36].

A further limitation concerns the device-specific nature of LDE, which is sensitive to IMU noise and sampling behaviour. Inertial sensor characteristics vary across smartphone models due to differences in Microelectromechanical Systems (MEMS) hardware, sampling rates, and operating system–level signal processing, meaning the raw-signal LDE values reported here may not compare directly to devices with different IMU hardware or sampling fidelity. Prior work has demonstrated such variability both between and within platforms, with the Android ecosystem exhibiting greater heterogeneity across devices than iOS [14], supporting the use of an iPhone in the present study. As findings are therefore specific to the iPhone SE 2020 running iOS 18, caution should be exercised when interpreting results in the context of other smartphone models [14]. Future work should quantify cross-device agreement using the same protocol across models; until then, studies should use a single phone model and operating system, report device details, and avoid applying absolute cut-offs without device-specific validation.

Our use of a 20 m walkway, due to physical constraints, differs from the 6MWT recommendation (≥30 m). The 6MWT is designed to measure submaximal functional exercise capacity. A 30 m walkway is recommended to maximize standardization of the test, specifically with respect to the impact of walkway length on distance achieved. However, we were not interested in distance as an outcome measure. We chose to use the 6MWT primarily for the standardization of instructions to participants. A shorter walkway could increase the proportion of time accelerating and decelerating. However, to minimise this impact we (1) removed 1.5 s before and after the turn and (2) for every lap we considered a full left step (based on VT and ML acceleration) to extract walking data, which in most cases made turn limits longer. Further, of all strides worth of data we used the middle 150 strides for optimal statistical precision [4]. It is also important to note that although 30 m is suggested, adaptations due to space limitations are acceptable in different clinical populations [41]. Hence, we believe that the conclusions regarding the aims of this paper, namely validity and age discriminability, are unlikely to have been affected by the choice of corridor length. However, caution should be taken when comparing our results to other evidence. Future work, e.g., to establish much needed normative values, should consider the benefits of a longer walkway (greater steady-state walking) versus a protocol that might be more feasible in more locations (e.g., clinics/homes), facilitating standardisation.

Finally, most previous studies report reduced gait stability in older adults, particularly in specific directions and when using 3D LDE. Our older adult sample, although community-dwelling, was largely recruited from a community centre with regular participation in structured exercise programs, which may have preserved dynamic stability and attenuated expected between-group differences relative to studies sampling more sedentary or frailer cohorts.

## 5. Conclusions

The LDE derived from an iPhone’s sensors (iPhone SE 2020) is a valid measure of walking stability in both young and older adults. We observed no between-group differences, likely because our older cohort was relatively fit and healthy and may have preserved dynamic stability. Overall, these findings support mobile phones as a practical tool for assessing walking stability, with potential for frequent self-assessment and as an outcome measure in clinical trials.

## Figures and Tables

**Table 1:** Participants demographics.

	Older Adults (n = 20)			Young Adults (n = 20)		
	Mean	SD	Range	Mean	SD	Range
Age (years)	76.4	4.66	68–85	27.15	6.56	21–42
Height (m)	1.64	0.09	1.50–1.95	1.70	0.11	1.50–1.91
Mass (kg)	69.96	12.26	50–105	68.9	10.59	50–86
BMI (kg/m^2^)	25.89	3.79	17.50–33.98	23.71	3.21	19.56–31.24

**Table 2:** Intra-class correlation coefficients (ICC) and 95% confidence intervals (CI) between wearable and mobile phone sensors overall and across 5 directions.

LDE	ICC	Lower CI	Upper CI	*p*-Value
Overall	0.844	0.567	0.926	<0.001
Vertical (VT)	0.829	−0.045	0.957	<0.001
Mediolateral (ML)	0.736	−0.068	0.924	<0.001
Anteroposterior (AP)	0.621	0.355	0.790	<0.001
Norm (N)	0.635	−0.057	0.894	<0.001
Three-dimensional (3D)	0.630	−0.740	0.887	<0.001

**Table 3:** Mean difference, relative mean difference, and LoAs between wearable and mobile sensors LDEs. LoA: limits of agreement. SD: standard deviation.

Direction	Mean Difference	SD	Relative Mean Difference (%)	Lower LoA	Upper LoA
Overall	0.081	0.039	1.027	0.003	0.158
Vertical (VT)	0.107	0.059	1.038	−0.009	0.225
Mediolateral (ML)	−0.055	0.126	0.978	−0.304	0.192
Anteroposterior (AP)	0.133	0.051	1.044	0.031	0.235
Norm (N)	0.101	0.048	1.043	0.006	0.195

## Data Availability

The raw data supporting the conclusions of this article will be made available by the authors on request.

## Abbreviations

The following abbreviations are used in this manuscript:

LDE	Local divergence exponent
VT	Vertical
ML	Mediolateral
AP	Anteroposterior
N	Norm
3D	Three-dimensional
IMU	Inertial measurement unit
MEMS	Microelectromechanical Systems
ICC	Intraclass correlation
OG	Older adults
YG	Young adults
LoA	Limits of agreement
